# Joint Optimization of Distribution Network Design and Two-Echelon Inventory Control with Stochastic Demand and CO_2_ Emission Tax Charges

**DOI:** 10.1371/journal.pone.0168526

**Published:** 2017-01-19

**Authors:** Shuangyan Li, Xialian Li, Dezhi Zhang, Lingyun Zhou

**Affiliations:** 1 School of Transportation and Logistics, Central South University of Forestry and Technology, Changsha, Hunan, China; 2 School of Traffic & Transportation Engineering, Central South University, Changsha, Hunan, China; 3 School of Management Engineering, Huaiyin Institute of Technology, Huaian, Jiangsu, China; Beihang University, CHINA

## Abstract

This study develops an optimization model to integrate facility location and inventory control for a three-level distribution network consisting of a supplier, multiple distribution centers (DCs), and multiple retailers. The integrated model addressed in this study simultaneously determines three types of decisions: (1) facility location (optimal number, location, and size of DCs); (2) allocation (assignment of suppliers to located DCs and retailers to located DCs, and corresponding optimal transport mode choices); and (3) inventory control decisions on order quantities, reorder points, and amount of safety stock at each retailer and opened DC. A mixed-integer programming model is presented, which considers the carbon emission taxes, multiple transport modes, stochastic demand, and replenishment lead time. The goal is to minimize the total cost, which covers the fixed costs of logistics facilities, inventory, transportation, and CO_2_ emission tax charges. The aforementioned optimal model was solved using commercial software LINGO 11. A numerical example is provided to illustrate the applications of the proposed model. The findings show that carbon emission taxes can significantly affect the supply chain structure, inventory level, and carbon emission reduction levels. The delay rate directly affects the replenishment decision of a retailer.

## Introduction

Sustainable supply chain management (SSCM) has become a hot topic for researchers and practitioners who aim to integrate environmental and social aspects with economic considerations [1,2]. Thus, managing and optimizing sustainable supply chains presents multiple challenges for many companies, among which the three important tasks are facility location, inventory control, and transport model decisions with consideration of CO_2_ emissions [3–5]. Being able to formulate a decision support system, including the aforementioned three decisions, is a major challenge that can result in a tremendous competitive advantage for a company in the market.

In this paper, the joint optimization of distribution network design and two-echelon inventory control with stochastic demand that considers CO_2_ emission tax charges was addressed to minimize the system-wide costs consisting of location, inventory, transportation, and carbon emission costs. The following issues have to be solved:

1. determining optimal locations and sizes of distribution centers (DCs);
2. allocating suppliers and retailers to the opened DCs;
3. setting the rational order quantity and reorder point for the ordered products at each opened DC and retailer; and
4. selecting the optimal combined transportation modes to define the balance between low carbon emission and high transportation cost.

The main innovative points of this study can be summarized as follows:

1. The inventory control decisions of retailers are considered. Most traditional two-echelon location–inventory problems neglect the inventory cost and the inventory control decisions of retailers. With the growing competition in the retail industry, cutting system-wide costs is essential to winning. Therefore, the inventory cost of retailers should be considered. Moreover, this study considers the stochastic replenishment lead time at the level of retailers.
2. Environmental factors are considered, and the costs of CO_2_ emissions are incorporated into the objectives. The two main activities considered were transportation and inventory management in relation to carbon emission sources. In particular, several alternative transportation modes exist from the supplier to the DCs, as well as from the DCs to the retailers. Furthermore, the effect of carbon emission tax policy on the structure of the distribution network is analyzed.
3. A two-echelon location–inventory model is established. In this model, several alternative size options exist for the candidate DCs. In addition, the capacity constraint in this study ensures that the maximum possible inventory accumulation at DCs does not exceed the size of the DCs.

The rest of this paper is organized as follows: Section 2 reviews the literature related to this problem. Section 3 presents the integrated inventory–location model. The computational results are illustrated in Section 4. Section 5 summarizes the conclusion and contributions of this study.

## Literature Review

SSCM mainly involves three decisions, namely, operational (short-term), tactical (medium-term), and strategic (long-term) decisions based on the time frame of decision making. One of the long-term strategic decision problems in supply chain management is distribution network design (DND). The DND problem can be classified into single-, two-, and multi-echelon models, depending on graph levels or echelons [6–8]. Wang et al. [9] investigated a two-echelon logistics distribution network design optimization model that is solved by a hybrid algorithm embedded with the particle swarm optimization (PSO) and genetic algorithm (GA). Moreover, facility location problem (FLP) is a classical model that solves the DND. For a more detailed review of integrated DND models, the reader is referred to the work of Lemmens et al. [8,10,11].

The location–inventory problem (LIP) is an extension of the classical facility location problem (FLP), which is also NP-hard [12,13]. LIP is basically an integrated discrete location problem that simultaneously determines location, allocation, and inventory decisions, which has received much attention in the past two decades. Chen and Ting [14] described a multiple ant colony system (MACS) to solve the single-source capacitated facility location problem (SSCFLP). Miranda and Garrido [15] addressed a joint location–distribution–inventory model for a three-layered supply chain, which is based on Lagrangian relaxation with consideration of the validity inequalities derived from the finite set of possible combinations of mean demand and variance. Liao et al. [16] presented a multi-objective location–inventory problem (MOLIP) model in vendor-managed inventory using non-dominated sorting genetic algorithm (NSGA-II). Tsao et al. [17] developed a continuous approximation approach to solve an integrated facility location–allocation and inventory management problem. Tancrez et al. [18] proposed a nonlinear continuous formulation that includes transportation, fixed, handling, and holding costs. Diabat et al. [19] considered a closed-loop location–inventory problem that is solved by a two-phase Lagrangian relaxation algorithm.

Compared with the traditional LIPs, the location–inventory model considering the stochastic environment is more practical. Shu and Sun [20] and Snyder et al. [21] addressed a robust location–inventory problem with uncertain demand at retailers. Shahabi et al. [22] studied a location–inventory problem in a three-level supply chain network with uncertainty parameters. Alhaj et al. [23] investigated a carbon-sensitive two-echelon inventory supply chain model with stochastic demand considering environmental impact.

As far as the inventory control decision problem is concerned, many studies have concentrated on the inventory management of DCs. Shen et al. [24] introduced a distribution center location model and proposed a heuristic algorithm based on Lagrangian relaxation. Cabrera et al. [25] solved a novel inventory–location model with a stochastic capacity constraint based on a periodic inventory control policy. Qu et al. [26] investigated a practical and novel location–inventory problem with stochastic demand considering different replenishment policies. Recently, two-echelon inventory control decisions have elicited concern. Teo et al. [27] addressed the two-echelon joint location–inventory model that arises from coordination of replenishment activities between warehouses and retailers in which the inventory control at both the DCs and retailers was considered. Uster et al. [28] proposed a continuous location–inventory problem with a power-of-two replenishment policy. Shu et al. [29] proposed a two-stage stochastic model to address the two-echelon location–inventory network design problem with uncertain demand in which the first stage decides the opening of warehouses and the second determines the warehouse–retailer assignments and two-echelon inventory replenishment strategies.

In relation to the inventory control decisions under multi-echelon distribution networks, previous studies mainly focused on replenishment time, order quantity, and safety stock at DCs or retailers, or both. However, limited studies have been conducted on the interaction between the inventory control decisions of DCs and retailers. The present study considers the inventory control decisions of retailers, which are usually relegated to the traditional LIP model.

The problem addressed in this study is also related to a class of the location–inventory problem with capacity restrictions. Romeijn et al. [30] investigated the two-echelon supply network optimization problem, which considers the capacity and congestion of DCs. Ozsen et al. [31] presented a location–inventory model subject to restriction on the maximum possible inventory accumulation at DCs. Miranda and Garrido [32] considered a capacitated location–inventory model with finite capacities of DCs. The aforementioned papers typically considered the capacity restrictions of candidate DCs but ignored the optimal size choices of candidate DCs.

The traditional LIP model mainly focuses on total costs or operator efficiency and only slightly considers transport mode choices and the corresponding external environmental costs. Kutanoglu and Mahajan [33] proposed an integrated optimization model to allocate stock levels at warehouses in a service part logistics network and considered two-echelon variable transportation costs, i.e., the costs of transporting the part from the central to the local warehouses and from the local warehouses to the customers. Luathep et al. [22] considered a three-level location–inventory problem in which the transportation cost per unit shipment, both between plants and warehouses and between warehouses and retailers, is assumed to be proportional to the Euclidean distance. Archetti et al. [34] addressed a closed-loop network with capacity limits, single-item management, and uncertainty on product demands and returns by a fuzzy programming method. Diabat et al. [35] addressed a joint location–inventory problem and extended it to account for the reduction of carbon emissions in which a three-level supply chain network is considered, i.e., one plant, multiple DCs, and multiple retailers. Wu et al. [36] proposed a multi-period location model with transportation economies of scale that distributes a single perishable product, as well as considerations of CO_2_ emissions on transport modes.

To the best of our knowledge, existing related studies integrating logistics network design and two-echelon inventory with the considerations of environment impacts are still scarce. The present work aims to fill this gap by focusing on the joint optimization of distribution network design (DND) and two-echelon inventory control (2-EIC) with stochastic demand and CO_2_ tax charges. First, an optimal programming model is proposed to address the simultaneous optimization problem of the selection of DCs, transport model choice and supply chain inventory control considering the CO_2_ emission taxes charges. Second, some management insights are obtained, which are based on the analysis of numerical experiments.

## Problem Formulation

### Problem description

**Fig 1:**
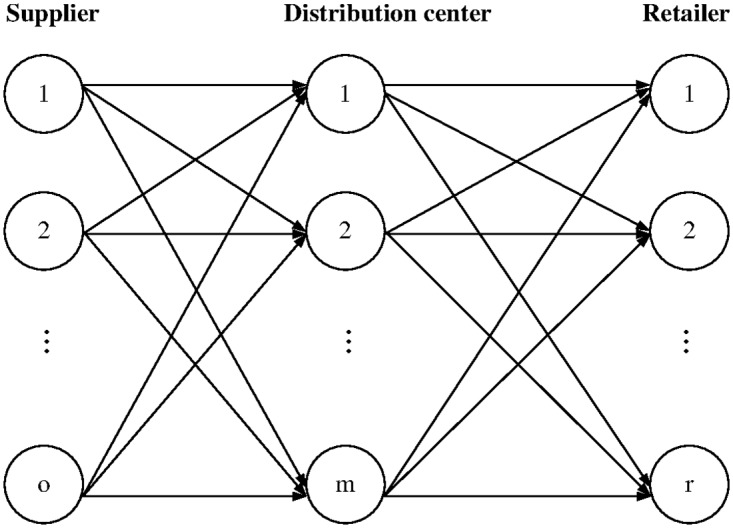
Illustration of proposed model.

The proposed problem is described as follows. A three-level distribution network consisting of multiple suppliers, DCs, and retailers is considered, as shown in Fig 1 The multiple suppliers serve a set of DCs, which in turn fulfill the demands at the locations of multiple retailers. Inventories are retained at the opened DCs and retailers. The (*R*, *Q*) inventory policy is applied to DCs and retailers. Under the (*R*, *Q*) inventory policy, an order of *Q* units is triggered when the inventory level falls below the reorder point *R*; thus, the resulting inventory position after ordering is in the interval [*R*, *R* + *Q*]. The replenishment lead times from suppliers to DCs are assumed constant. However, the replenishment lead times from DCs to retailers are stochastic, considering the effects of supply shortages at DCs.

In this study, the integrated inventory and location model based on the principle of system-wide cost minimization considering the candidate DC’s capacity is investigated.

The integrated optimization model in this paper simultaneously determines three types of decisions: (1) facility location, i.e., the number, location, and size of DCs; (2) allocation, i.e., assignment of suppliers to located DCs and retailers to located DCs, as well as the corresponding optimal transport mode selection; and (3) inventory control decisions on the order quantities, reorder points, and amount of safety stock at each retailer and opened DC. The goal is to minimize the total costs that comprise the facility location, transportation, inventory, and carbon emission tax charges.

### Assumptions

To facilitate the presentation of essential ideas without loss of generality, the following basic assumptions are made:

**A1** The demand of each customer is stochastic and independent, which follows a normal distribution with known mean and variance.
**A2** The replenishment lead time of DCs is constant. The replenishment time of retailers is uncertain.
**A3** A single sourcing strategy is adopted in which each DC is assigned to a single supplier, and each retailer is assigned to a single DC.
**A4** DCs and retailers adopt a continuous inventory policy (*R*, *Q*).
**A5** Shortages are allowed and result in unfulfilled demand cost.
**A6** Transportation cost includes two parts. The variable transportation costs are linearly proportional to the product of the quantity of products and traveling distances. The fixed transportation cost is also considered, which is used to identify the fixed operator cost per shift of different transport tools.
**A7** At each DC and each retailer, CO_2_ emissions are linearly proportional to the quantity of products processed. In transportation, carbon emissions are linearly proportional to the product of the quantity of products and traveling distances.
**A8** The replenishment lead time of retailers includes two parts, i.e., transport and delay times caused by absence of stock at DCs. We assume that the delay time is proportional to the amount of shortages that occurred at DCs. Moreover, the replenishment lead time of retailers is mainly affected by the delay time caused by out-of-stock products at DCs. Thus, in this study, the effect of transport time on the replenishment lead time of retailers can be ignored.

### Notations

**Indices**
*o*: index for suppliers (1,⋯,*s*)
*l*: index for transport modes (1,⋯,*w*)
*i*: index for DCs to be set up in candidate sites (1,⋯,*m*)
*k*: index for retailers (1,⋯,*r*)
*a*: index for sizes available to DCs (1,⋯,*b*)

#### Parameters

**Parameters associated with DCs**
*D*_*i*_: demand rate for product per time unit at DC *i*
$u_{i}^{\prime }$: mean demand per time unit of DC *i*
$\sigma_{i}^{\prime }$: standard deviation of demand per time unit of DC *i*
$u_{i}^{\prime }\left(LT_{i}\right)$: average demand at DC *i* during replenishment lead time
$\sigma_{i}^{\prime }\left(LT_{i}\right)$: standard deviation of demand at DC *i* during replenishment lead time
*CT*_*oli*_: unit transportation cost from supplier *o* to DC *i* by transport mode *l*
*SC*_*ol*_: fixed transportation cost from supplier *o* to DCs by transport mode *l*
*C*_*ia*_: capacity with level *a* for DC *i*
*H*_*i*_: daily inventory holding cost per unit of product at DC *i*
*P*_*i*_: unit penalty cost for unfilled demand at DC *i*
*LQ*_*i*_: unfulfilled demand at DC *i*
*OR*_*i*_: ordering cost at DC *i*
*LT*_*i*_: replenishment lead time for DC *i*
*L*_*oi*_: distance between supplier *o* and DC *i*
*EV*_*oli*_: per-unit CO_2_ emission to ship from supplier *o* to DC *i* by transport mode *l*
*EF*_*i*_: per-unit CO_2_ emission to process within DC *i*
*k*_*i*_: level of service linked to safety stock level at DC *i*
*δ*: level of service linked to inventory capacity constraint at DCs

**Parameters associated with retailers**
*d*_*k*_: demand rate for product per time unit at retailer *k*
*u*_*k*_: mean demand per time unit at retailer *k*
*σ*_*k*_: standard deviation of demand per time unit at retailer *k*
${\tilde{u}}_{k}$: average demand at retailer *k* during replenishment lead time
${\tilde{\sigma }}_{k}$: standard deviation of demand at retailer *k* during replenishment lead time
*ct*_*ilk*_: unit transportation cost from DC *i* to retailer *k* by transport mode *l*
*sc*_*il*_: fixed transportation cost from DC *i* to retailers by transport mode *l*
*f*_*ia*_: fixed cost of opening and operating a DC at location *i* with size *a* per unit time
*h*_*k*_: daily inventory holding cost per unit of product at retailer *k*
*p*_*k*_: unit penalty cost for unfilled demand at retailer *k*
*lq*_*k*_: unfulfilled demand at retailer *k*
*or*_*k*_: ordering cost at retailer *k*
*l*_*ik*_: distance between DC *i* and retailer *k*
*e*: carbon tax rate
*t*_*ilk*_: transportation time from DC *i* to retailer *k* by transport mode *l*
*ev*_*ilk*_: per-unit CO_2_ emission to ship from DC *i* to retailer *k* by transport mode *l*
*ef*_*k*_: per-unit CO_2_ emission to process at retailer *k*
*u*(*lt*_*k*_): average replenishment lead time at retailer *k*
*σ*(*lt*_*k*_): standard deviation of replenishment lead time at retailer *k*
*λ*: delay rate, which is used to evaluate the effects of shortages at DCs to replenishment lead time of retailers
*W*: upper bound of order quantity of retailers

**Parameters associated with costs**
*FDC*: fixed cost for opening and operating DCs per unit time
*OC*_*i*_: expected ordering cost of DC *i* per unit time
*AQ*_*i*_: average stock quantity of DC *i*
*HDC*_*i*_: expected inventory holding cost per unit time at DC *i*
*PDC*_*i*_: shortage cost during replenishment lead time at DC *i*
*TDCH*_*i*_: expected replenishment cost per unit time at DC *i*
*oc*_*k*_: expected ordering cost per unit time at retailer *k*
*hc*_*k*_: expected inventory holding cost per unit time at retailer *k*
*pc*_*k*_: shortage cost per unit time at retailer *k*
E(·), Var(·): representing the corresponding mean and variance functions respectively
*HRE*_*k*_: expected replenishment cost per unit time at retailer *k*
*TRFC*: transportation cost per unit time
*EMC*: expected carbon emissions cost per unit time
*Z*: total cost of distribution network per unit time

**Decision variables**
$x_{oli}^{\prime }$: binary variable, which takes the value of 1 if DC *i* is assigned to supplier *o* by transport mode *l*, and 0 otherwise
*x*_*ilk*_: binary variable, which takes the value of 1 if DC *i* is assigned to retailer *k* by transport mode *l*, and 0 otherwise
*u*_*ia*_: binary variable, which takes the value of 1 if DC *i* is opened with size *a*, and 0 otherwise
*R*_*i*_: reorder point at DC *i*
*r*_*k*_: reorder point at retailer *k*
*Q*_*i*_: order quantity at DC *i* from a certain supplier
*q*_*k*_: order quantity at retailer *k* from a certain DC
*lt*_*k*_: replenishment lead time at retailer *k*

### Cost analysis

#### Fixed cost for opening and operating DCs

The fixed cost for opening and operating DCs per unit time is calculated by Eq (1).

$$FDC=\overset{m}{\underset{i=1}{\sum }}\overset{b}{\underset{a=1}{\sum }}f_{ia}u_{ia}.$$ (1)

#### Replenishment costs of DCs

The replenishment costs considered in this study comprise ordering, inventory holding, and shortage costs.

(1) Ordering cost

The expected ordering cost of DC *i* per unit time is expressed as follows:

$$OC_{i}=OR_{i}\frac{u_{i}^{\prime }}{Q_{i}}.$$ (2)

(2) Inventory holding cost

Given that the demand of each DC is a linear superposition function of customer demand, which is normally distributed and independent, the demand rate of DC *i* is $D_{i}=\sum_{l=1}^{w}\sum_{k=1}^{r}x_{ilk}d_{k}\;$, the mean of demand per unit time at DC *i* is $u_{i}^{\prime }=\sum_{l=1}^{w}\sum_{k=1}^{r}x_{ilk}u_{k}\;$, and the variance of demand per unit time at DC *i* is ${\left(\sigma_{i}^{\prime }\right)}^{2}=\sum_{l=1}^{w}\sum_{k=1}^{r}x_{ilk}^{\text{2}}{\left(\sigma_{k}\right)}^{2}$. Thus, the demand at DC *i* in each replenishment interval of *LT*_*i*_ is a normal distribution with a mean of ${\tilde{u}}_{i}=LT_{i}\sum_{l=1}^{w}\sum_{k=1}^{r}x_{ilk}u_{k}$ and variance of ${\left({\tilde{\sigma }}_{i}\right)}^{2}=LT_{i}\sum_{l=1}^{w}\sum_{k=1}^{r}x_{ilk}^{\text{2}}{\left(\sigma_{k}\right)}^{2}$. The reorder point of DC *i* with the *κ*_*i*_ level of service, which is linked to the safety stock level, is $R_{i}=LT_{i}u_{i}^{\prime }+Z_{\kappa_{i}}\sqrt{LT_{i}}\sigma_{i}^{\prime }$ (see Miranda and Garrido [37]), where $Z_{\kappa_{i}}$ is the value of cumulative standard normal distribution up to a *κ*_*i*_ probability.

This study assumes that DCs adopt a continuous inventory policy (*R*, *Q*). In many traditional studies in the existing literature, the order quantity is obtained by the first-order optimality conditions of the replenishment cost function. Therefore, the order quantity is no longer variable and can be eliminated from the model. By contrast, this procedure would be invalid if any constraint relies on the order quantity. In the model of the present study, a constraint dependent on the order quantity was included. Thus, an alternative procedure to solve the resulting model was presented.

**Fig 2:**
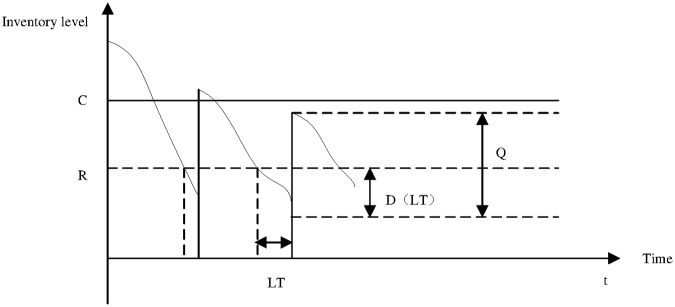
Illustration of inventory control policy with inventory capacity constraint.

The capacity restriction of DCs is not measured by the mean of each DC demand. The constraint declares a maximum inventory level for each DC based on chance-constrained programming. Given a fixed number *δ*, the probability of the inventory level of DC at peak times exceeds its capacity as shown in Fig 2 This probability is known as the level of service for the inventory capacity constraint at each DC. Consequently, this inventory level corresponds to the reorder point minus the stochastic demand during the replenishment lead time, plus the replenishment order quantity. Thus, the inventory capacity constraint can be written as follows:

$$\text{Pr}(R_{i}-D(LT_{i})+Q_{i}\geq C_{ia}u_{ia})=\delta .$$ (3)

The reorder point of DC *i* with the level of service linked to the safety stock level *κ*_*i*_ is $R_{i}=LT_{i}u_{i}^{\prime }+Z_{\kappa_{i}}\sqrt{LT_{i}}\sigma_{i}^{\prime }$. Therefore, Eq (3) can be reformulated as follows:

$$\text{Pr}(R_{i}-D(LT_{i})+Q_{i}\leq C_{ia}u_{ia})=1-\delta .$$ (4)

Thus, this constraint can be expressed as follows (details of the derivation process of Eq (5) are provided in Appendix A1 in S1 File):

$$Q_{i}+Z_{\kappa_{i}}\sqrt{LT_{i}}\sigma_{i}^{\prime }+Z_{1-\delta }\sqrt{LT_{i}}\sigma_{i}^{\prime }\leq C_{ia}u_{ia},$$ (5)

where *Z*_1‒*δ*_ is the value of the standard normal distribution, which accumulates a probability of 1 ‒ *δ*.

The average stock quantity of DC *i* can be expressed as follows:

$$AQ_{i}=\frac{Q_{i}}{2}+Z_{\kappa_{i}}\sqrt{LT_{i}}\sigma_{i}^{\prime }.$$ (6)

where *k*_*i*_ is called a ‘‘service level”, which means that stockouts occur during the lead time with a probability of *k*_*i*_ or less, and $z_{k_{i}}$ is the standard normal deviation such that $P(Z\leq z_{k_{i}})=k_{i}$ [38]. For example, if *k*_*i*_ = 0.025, which means the lead time demands are satisfied with a probability of 97.5%, $z_{k_{i}}\;=\;1.96$.

Therefore, the expected inventory holding cost per unit time at DC *i* can be written as follows:

$$HDC_{i}=H_{i}(\frac{Q_{i}}{2}+Z_{\kappa_{i}}\sqrt{LT_{i}}\sigma_{i}^{\prime }).$$ (7)

(3) Shortage cost

Under a continuous review inventory policy (*R*, *Q*), the stock-out phenomenon occurs only when the cumulative demand during the replenishment lead time exceeds the maximum inventory level *R* at that particular time. Thus, the expected unfulfilled demand at DC *i* can be computed as follows (details of the derivation process of Eq (8) are provided in Appendix A2 in S1 File):

$$\text{E}(LQ_{i})=\overset{+\infty }{\underset{R_{i}}{\int }}(x-R_{i})f(x)dx=\sqrt{LT_{i}}\sigma_{i}^{\prime }(\phi (\kappa_{i})+\kappa_{i}\Phi (\kappa_{i})-\kappa_{i}),$$ (8)

where $\kappa_{i}=\frac{\left(R_{i}-LT_{i}u_{i}^{\prime }\right)}{\sqrt{LT_{i}}\sigma_{i}^{\prime }}$, $\Phi (\kappa_{i})=\int_{-\infty }^{\kappa_{i}}\phi \left(x\right)dx$, $\phi \left(\kappa_{i}\right)=\frac{1}{\sqrt{2\pi }}e^{\frac{-\kappa_{i}^{2}}{2}}$.

In addition, the variance of unfulfilled demand at DC *i* can be computed as follows (details of the derivation process of Eq (9) are provided in Appendix A3 in S1 File):

$$\text{Var}(LQ_{i})=LT_{i}{\left(\sigma_{i}^{\prime }\right)}^{2}[1+\kappa_{i}\phi (\kappa_{i})-\Phi (\kappa_{i})+{\left(\kappa_{i}\right)}^{2}\Phi (\kappa_{i})-{\left(\kappa_{i}\right)}^{2}{\left(\Phi (\kappa_{i})\right)}^{2}-{\left(\phi (\kappa_{i})\right)}^{2}-2{\left(\kappa_{i}\right)}^{2}\phi (\kappa_{i})\Phi (\kappa_{i})].$$ (9)

Therefore, the shortage cost during the replenishment lead time at DC *i* is as follows:

$${\text{PDC}}_{i}={\text{PC}}_{i}(\sqrt{{\text{LT}}_{i}}\sigma_{i}^{\prime }(\phi (\kappa_{i})+\kappa_{i}\Phi (\kappa_{i})-\kappa_{i})).$$ (10)

The expected replenishment cost per unit time at DC *i* is formulated as follows:

$${\text{TDCH}}_{i}={\text{OR}}_{i}\frac{u_{i}^{\prime }}{Q_{i}}+H_{i}(\frac{Q_{i}}{2}+Z_{\kappa_{i}}\sqrt{\text{L}{\text{T}}_{i}}\sigma_{i}^{\prime })+{\text{PC}}_{i}(\sqrt{\text{L}{\text{T}}_{i}}\sigma_{i}^{\prime }(\phi (\kappa_{i})+\kappa_{i}\Phi (\kappa_{i})-\kappa_{i}))\frac{u_{i}^{\prime }}{Q_{i}}.$$ (11)

#### Replenishment costs of retailers

The stochastic demand of each retailer follows a normal demand distribution. The replenishment lead time at retailers is assumed to be the transportation time from DCs to retailers plus the time delays caused by the shortages at DCs. Thus, **$lt_{k}=\sum_{i=1}^{m}\sum_{l=1}^{w}x_{ilk}t_{ilk}+\lambda \sum_{i=1}^{m}\sum_{l=1}^{w}x_{ilk}LQ_{i}$**. According to assumption A8, $lt_{k}=\lambda \sum_{i=1}^{m}\sum_{l=1}^{w}x_{ilk}LQ_{i}$ can be obtained. Therefore, the expected replenishment lead time at retailer *k* can be expressed as follows:

$$\text{E}\left(lt_{k}\right)=u(lt_{k})=\text{E}\left(\lambda \sum_{i=1}^{m}\sum_{l=1}^{w}x_{ilk}LQ_{i}\right)=\lambda \sum_{i=1}^{m}\sum_{l=1}^{w}x_{ilk}^{}E(LQ_{i})$$ (12)

The variance of the replenishment lead time at retailer *k* can be expressed as follows:

$$\text{Var}(lt_{k})={\left(\sigma (lt_{k})\right)}^{2}=\text{Var}(\lambda \overset{m}{\underset{i=1}{\sum }}\overset{w}{\underset{l=1}{\sum }}x_{ilk}LQ_{i})=\lambda^{2}\overset{m}{\underset{i=1}{\sum }}\overset{w}{\underset{l=1}{\sum }}x_{ilk}^{2}\text{Var}(LQ_{i}).$$ (13)

Thus, the demand at retailer *k* in each replenishment interval of *lt*_*k*_ is a normal distribution with a mean of ${\tilde{u}}_{k}=u\left(lt_{k}\right)u_{k}$ and standard deviation of ${\tilde{\sigma }}_{k}=\sqrt{u\left(lt_{k}\right){\left(\sigma_{k}\right)}^{2}+{\left(u_{k}\right)}^{2}{\left(\sigma \left(lt_{k}\right)\right)}^{2}},$ where E(·) and Var(·) represent the corresponding mean and variance functions, respectively.

(1) Ordering cost

The expected ordering cost per unit time at retailer *k* is expressed as follows:

$$oc_{k}=or_{k}\frac{{\tilde{u}}_{k}}{q_{k}}.$$ (14)

(2) Inventory holding cost

The expected inventory holding cost per unit time at retailer *k* is expressed as follows:

$$hc_{k}=h_{k}(\frac{q_{k}}{2}+Z_{\tau_{k}}\sqrt{lt_{k}}{\tilde{\sigma }}_{k}).$$ (15)

(3) Shortage cost

Similar to the derivation process of the expected unfulfilled demand of DCs, the expected unfulfilled demand at retailers can be expressed as follows:

$$\text{E}\left(lq_{k}\right)=\int_{r_{k}}^{+\infty }\left(x-r_{k}\right)g_{k}\left(x\right)dx={\tilde{\sigma }}_{k}\left(\phi \left(\tau_{k}\right)+\tau_{k}\Phi \left(\tau_{k}\right)-\tau_{k}\right),$$ (16)

where $g_{k}\left(x\right)=\frac{1}{\sqrt{2\pi }{\tilde{\sigma }}_{k}}e^{-\frac{{\left(x-{\tilde{u}}_{k}\right)}^{2}}{2{\left({\tilde{\sigma }}_{k}\right)}^{2}}}$ and $\tau_{k}=\frac{r_{k}-{\tilde{u}}_{k}}{{\tilde{\sigma }}_{k}}$.

Therefore, the shortage cost per unit time at retailer *k* is calculated as follows:

$${\text{pc}}_{k}=p_{k}\left({\tilde{\sigma }}_{k}\left(\phi \left(\tau_{k}\right)+\tau_{k}\Phi \left(\tau_{k}\right)-\tau_{k}\right)\right)\frac{{\tilde{u}}_{k}}{q_{k}}.$$ (17)

The expected replenishment cost per unit time at retailer *k* can be expressed as follows:

$$HRE_{k}={\text{or}}_{k}\frac{{\tilde{u}}_{k}}{q_{k}}+h_{k}(\frac{q_{k}}{2}+r_{k}-{\tilde{u}}_{k})+p_{k}\left({\tilde{\sigma }}_{k}\left(\varphi \left(\tau_{k}\right)+\tau_{k}\Phi \left(\tau_{k}\right)-\tau_{k}\right)\right)\frac{{\tilde{u}}_{k}}{q_{k}}.$$ (18)

#### Transportation costs

Transportation costs include the variable and fixed transportation costs. The two-echelon variable transportation costs include two parts, i.e., inbound network transportation cost (cost of shipping the products from the suppliers to the DCs) and outbound network transportation cost (cost of shipping the products from the DCs to retailers). Furthermore, the fixed costs of transportation are independent of the quantity of products and shipping distances. Thus, the transportation cost per unit time can be formulated as follows:

$$\text{TRFC}=\sum_{o=1}^{s}\sum_{i=1}^{m}\sum_{l=1}^{w}x_{{}_{oli}}^{\prime }SC_{ol}\frac{u_{i}^{\prime }}{Q_{i}}+\sum_{i=1}^{m}\sum_{l=1}^{w}\sum_{k=1}^{r}x_{ilk}sc_{il}\frac{{\tilde{u}}_{k}}{q_{k}}+\sum_{i=1}^{m}\sum_{o=1}^{s}\sum_{l=1}^{w}x_{{}_{oli}}^{\prime }u_{i}^{\prime }CT_{oli}L_{oi}+\sum_{i=1}^{m}\sum_{l=1}^{w}\sum_{k=1}^{r}x_{ilk}u_{k}ct_{ilk}L_{ik}.$$ (19)

#### Carbon emission costs

The costs of CO_2_ emission tax charges into the objectives were incorporated with consideration of the sustainability of the distribution network. Under the carbon emission tax policy, no strict cap is applied to carbon emissions, but the total carbon emissions will be penalized under a carbon tax policy. The tax is a financial penalty, which is assumed to be equal to the product of the total carbon emissions and the carbon tax rate. With regard to the carbon emission sources, two main activities were considered: transportation and inventory management. Furthermore, the average shipment from supplier *o* to DC *i* was calculated by mode *l* using the corresponding expected demand of DC *i* (i.e., $u_{i}^{\prime }$), while the average shipment from DC *i* to retailer *k* by mode *l* is expressed with the expected demand of retailer *k* (i.e., *u*_*k*_). Therefore, the expected total carbon emission cost per unit time can be calculated as follows:

$$\text{EMC}=\sum_{i=1}^{m}EF_{i}\left(\frac{Q_{i}}{2}+Z_{\kappa_{i}}\sqrt{LT_{i}}\sigma_{i}^{\prime }\right)+\sum_{k=1}^{v}ef_{k}\left(\frac{q_{k}}{2}+Z_{\tau_{k}}\sqrt{lt_{k}}{\tilde{\sigma }}_{k}\right)+\sum_{o=1}^{s}\sum_{l=1}^{w}\sum_{i=1}^{m}EV_{oli}x_{oli}^{\prime }u_{i}^{\prime }L_{oi}+\sum_{i=1}^{m}\sum_{l=1}^{w}\sum_{k=1}^{r}ev_{ilk}x_{ilk}u_{k}l_{ik}.$$ (20)

### Model formulation

Based on the preceding analysis, the formulation of the integrated inventory and location models was presented, which determines the optimal location and size of DCs, as well as the optimal order quantity and reorder point for the opened DCs and retailers. The model is formulated as follows:

$$\begin{array}{lll} \text{Min}Z & = & \sum_{i=1}^{m}\sum_{a=1}^{b}u_{ia}f_{ia}\\ & & +\sum_{i=1}^{m}\left(OR_{i}\frac{u_{i}^{\prime }}{Q_{i}}+H_{i}\left(\frac{Q_{i}}{2}+Z_{\kappa_{i}}\sqrt{LT_{i}}\sigma_{i}^{\prime }\right)+P_{i}\left(\sqrt{LT_{i}}\sigma_{i}^{\prime }\left(\phi \left(\kappa_{i}\right)+\kappa_{i}\Phi \left(\kappa_{i}\right)-\kappa_{i}\right)\right)\frac{u_{i}^{\prime }}{Q_{i}}\right)\\ & & +\sum_{k=1}^{v}\left(or_{k}\frac{{\tilde{u}}_{k}}{q_{k}}+h_{k}\left(\frac{q_{k}}{2}+Z_{\tau_{k}}\sqrt{lt_{k}}{\tilde{\sigma }}_{k}\right)+p_{k}\left({\tilde{\sigma }}_{k}\left(\varphi \left(\tau_{k}\right)+\tau_{k}\Phi \left(\tau_{k}\right)-\tau_{k}\right)\right)\frac{{\tilde{u}}_{k}}{q_{k}}\right)\\ & & +\left(\sum_{i=1}^{m}\sum_{l=1}^{w}z_{il}SC_{il}\frac{u_{i}^{\prime }}{Q_{i}}+\sum_{l=1}^{w}\sum_{k=1}^{r}y_{lk}sc_{lk}\frac{{\tilde{u}}_{k}}{q_{k}}+\sum_{i=1}^{m}\sum_{o=1}^{s}\sum_{l=1}^{w}x_{{}_{oli}}^{\prime }u_{i}^{\prime }CT_{oli}L_{oi}+\sum_{i=1}^{m}\sum_{l=1}^{w}\sum_{k=1}^{r}x_{ilk}u_{k}ct_{ilk}L_{ik}\right)\\ & & +e\left(\sum_{i=1}^{m}EF_{i}\left(\frac{Q_{i}}{2}+R_{i}-\mu_{i}^{\prime }LT_{i}\right)+\sum_{k=1}^{v}ef_{k}\left(\frac{q_{k}}{2}+r_{k}-{\tilde{u}}_{k}\right)\right)\\ & & +e\left(\sum_{i=1}^{m}\sum_{o=1}^{s}\sum_{l=1}^{w}x_{{}_{oli}}^{\prime }u_{i}^{\prime }EV_{oli}L_{oi}+\sum_{i=1}^{m}\sum_{l=1}^{w}\sum_{k=1}^{r}x_{ilk}u_{k}ev_{ilk}L_{ik}\right) \end{array}\;,$$ (21)

s.t.

$$u_{i}^{\prime }=\sum_{l=1}^{w}\sum_{k=1}^{r}x_{ilk}u_{k}\;\forall i,$$ (22)

$${\left(\sigma_{i}^{\prime }\right)}^{2}=\sum_{l=1}^{w}\sum_{k=1}^{r}x_{ilk}{\left(\sigma_{k}\right)}^{2}\;\forall i,$$ (23)

$$\sum_{i=1}^{n}\sum_{l=1}^{w}x_{ilk}=1\;\forall k,$$ (24)

$$\sum_{a=1}^{b}u_{ia}\leq 1\;\forall i,$$ (25)

$$\sum_{1=1}^{w}x_{ilk}\leq 1\;\forall i,k,$$ (26)

$$\sum_{l=1}^{w}x_{{}_{oli}}^{\prime }\leq 1\;\forall o,i,$$ (27)

$$q_{k}\leq W\;\forall k,$$ (28)

$$Q_{i}+Z_{\kappa_{i}}\sqrt{LT_{i}}\sigma_{i}^{\prime }+Z_{\delta }\sqrt{LT_{i}}\sigma_{i}^{\prime }\leq u_{ia}C_{ia}\;\forall i\;,$$ (29)

$$\sum_{o=1}^{s}\sum_{l=1}^{w}x_{oli}^{\prime }\geq \sum_{a=1}^{b}{\text{u}}_{ia}\;\forall i,$$ (30)

$$\sum_{a=1}^{b}{\text{u}}_{ia}\geq \sum_{1=1}^{w}x_{ilk}\;\forall i,k,$$ (31)

$$x_{ilk}\leq y_{lk}\;\forall i,l,k,$$ (32)

$$x_{oil}^{\prime },x_{ijk},u_{ia}\in \left\{0,1\right\}\;\forall o,a,i,j,k,$$ (33)

$$q_{k},Q_{i},R_{i},r_{k},lt_{k},\kappa_{i},\tau_{k}\geq 0\;\forall i,k.$$ (34)

The objective function of Eq (21) represents the total distribution network costs. The first term is the fixed cost of DCs. The second represents the fixed order, inventory holding, and penalty costs for unfilled demand at DCs, while the third term is that of retailers. The fourth term includes the total transport costs for inbound and outbound network transportation. The fifth and sixth terms are CO_2_ emission tax charging costs of inventory and transport process, respectively.

Constraints (22) and (23) represent the mean and variance of each DC demand. Constraint (24) ensures that each retailer is served by exactly one distribution center by only one transport mode. Constraint (25) implies that the candidate DCs select an option size from the alternative size set. Constraints (26) and (27) ensure that if two points on one echelon are related to each other, then one mode of transportation must be shared between them. Constraint (28) ensures that the order quantity of retailers is less than the corresponding upper bound. Constraint (29) states the capacity restriction for each DC. Constraint (30) implies that if DC *i* is opened, it must be serviced by a certain supplier. Constraint (31) ensures that only when DC *i* is opened can it serve retailers using various transport modes. Constraint (32) represents the transport mode selection constraints between inbound and outbound networks. Constraint (33) enforces that location decision and transport mode selection decision variables are binary. Finally, constraint (34) specifies that other decision variables are non-negative.

## Numerical Experiments

The proposed model and solution algorithm are applied to a simple three-echelon distribution network, which comprises a single supplier, three candidate DCs with three alternative sizes, and ten retailers. Three alternative transport modes can be chosen between supplier and DCs, as well as between DCs and retailers.

### Data input

The corresponding parameters are the following: (1) *e* (carbon emission tax rate) = 30, (2) *λ* (delay rate) = 0.15, (3) *LT* (replenishment lead time at DCs) = 1, (4) 1 − *δ* (level of service) = 0.95, (5) *W* (upper bound of order quantity of retailers) = 25. The suppliers, candidate DCs, and retailers are located randomly in an 800 km^2^ area based on a uniform distribution. Other parameters are shown in Table 1.

**Table 1:** Basic input parameters of numerical example.

Parameters	Values
Mean daily demand of retailer *k* (ton)	*u*_*k*_ = *U*[5,20]
Daily demand standard deviation of retailer *k* (ton)	*σ*_*k*_ = *U*[1,5]
Three alternative size of DCs (ton/per time unit)	*C* = (100,130,200}
Fixed cost of opening and operating a DC with different sizes at site *i* ($)	*f*_*ia*_ = {4,000, 5,000, 6,000} *U*[0.9,1.1]
Ordering cost at DC *i* ($)	OR_*i*_ = U[3,200, 3,800]
Ordering cost at retailer *k* ($)	*or*_*k*_ = U[1,000, 1,400]
Holding cost per ton per time unit at DC *i* ($)	*H*_*i*_ = *U*[18,20]
Holding cost per ton per time unit at retailer *k* ($)	*h*_*k*_ = *U*[8,13]
Unit penalty of shortage per time unit at DC *i*($)	*P_i_* = *U*[8,9]
Unit penalty of shortage per time unit at retailer *k* ($)	*p*_*k*_ = *U*[8,10]
Carbon emission per unit shipment to deal with within DC (ton/ton)	EF_*i*_ = *U*[0.8.1]
Carbon emission per unit shipment to deal with within retailer *k* (ton/ton)	ef_*k*_ = *U*[0.4.0.7]
Carbon emission per unit turnover from supplier *o* to DC *i* by mode *l* (ton/ton-km)	EV_*oli*_ = *U*[0.1,0.12]
Carbon emission per unit turnover from DC *i* to retailer *k* by mode *l* (ton/ton-km)	*ev*_*ilk*_ = U[0.06,0.09]
Fixed transportation cost from supplier *o* to DC *i* by mode *l* ($/shift)	SC_*oli*_ = *U*[200.500]
Fixed transportation cost from DC *i* to retailer *k* by mode *l* ($/shift)	*sc*_*ilk*_ = *U*[100,300]
Unit transportation cost from supplier *o* to DC *i* by mode *l* ($/ton-km)	*CT*_*oli*_ = *U*[1,1.2]
Unit transportation cost from DC *i* to retailer *k* by mode *l* ($/ton-km)	*ct*_*ilk*_ = *U*[0.8,1]

### Effect of carbon emission tax rate

This section studies the effect of carbon emission tax rate on distribution network design, inventory control decisions, and total cost invested. Six scenarios were considered with different carbon emission taxes to analyze the corresponding results. From scenarios 1 to 6, the carbon emission tax charges are set to 10, 20, 30, 40, 50, and 60 $/ton, respectively.

We first analyze the effects of carbon emission tax rate on location and inventory decisions of the distribution network. The results in Table 2 show that the total CO_2_ emissions of distribution network gradually decreases from 154.68 tons to 116.40 tons when the carbon emission tax rate increases from $10/ton to $60/ton. However, the total cost of the entire distribution network increases, thereby implying that the decrease range of carbon emissions is insufficiently large to eliminate the effect of the carbon tax rate increase on the carbon emission cost.

**Table 2:** Effects of carbon tax rate on performances of distribution network.

Carbon tax rate ($/ton)	10	20	30	40	50	60
Fixed cost ($)	5,400.00	5,100.00	5,100.00	5,100.00	5,400.00	5,400.00
Carbon emissions (ton)	154.68	147.38	138.15	136.59	121.48	116.40
Carbon emission cost ($)	1,546.80	2,947.60	4,144.50	5,463.60	6,074.00	6,984.00
Transportation cost ($)	12,329.50	13,493.00	13,493.00	13,518.60	12,329.50	12,329.50
Replenishment cost at retailers ($)	7,965.84	8,068.80	8,203.27	8,355.79	8,518.87	8,688.10
Replenishment cost at DCs ($)	5,394.33	5,608.02	5,702.65	5,810.56	5,743.69	5,852.95
Total cost ($)	32,636.56	35,217.55	36,643.50	38,248.82	38,066.24	39,255.04

**Fig 3:**
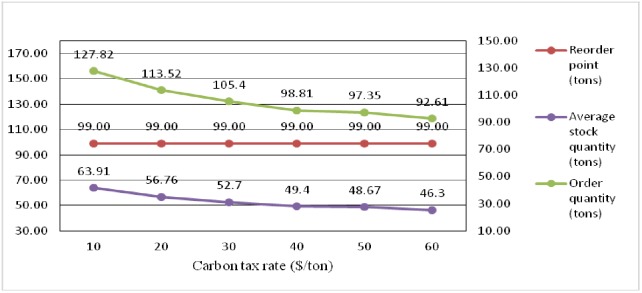
Comparative results of reorder point and order quantity and average shortage of opened DCs under different carbon tax rates.

Tables 3 and 4 indicate the effects of the carbon emission tax rate on the inventory control parameters of the opened DCs and retailers, respectively. Fig 3 shows that the order point of DCs remains the same and that the order quantity continuously decreases. The results presented in Table 3 indicate that the replenishment lead time and order point of retailers remain the same. Moreover, the order quantity in DCs continuously decreases from 127.82 tons to 92.61 tons with the increase of the carbon tax rate from 10$/ton to 60$/ton. Moreover, the order quantity of retailers continuously decreases with the increase in tax rate as shown in Table 4. Decreasing the average stock level is reasonable to decrease the total carbon emission of the entire distribution network, especially with the increase of the carbon tax rates.

**Table 3:** Comparative results of DCs under different carbon tax rates.

Carbon tax rate ($/ton)	10	20	30	40	50	60
Reorder point	(0, 99.00, 0)	(0, 0, 99.00)	(0, 0, 99.00)	(0, 0, 99.00)	(0, 99.00, 0)	(0, 99.00, 0)
Order quantity	(0, 127.82, 0)	(0, 0, 113.52)	(0, 0, 105.40)	(0, 0, 98.81)	(0, 97.35, 0)	(0, 92.61, 0)
Average stock quantity	(0, 63.91, 0)	(0, 0, 56.76)	(0, 0, 52.70)	(0, 0, 49.40)	(0, 48.67, 0)	(0, 46.30, 0)

Note: Expressions in brackets correspond to the solution of DCs in sequence.

**Table 4:** Comparative results of retailers under different carbon tax rates.

Scenarios	Carbon tax rate ($/ton)	Optimal solutions		
		Replenishment lead time	Reorder point	Order quantity
Scenario 1	10	(0.39, 0.39, 0.39, 0.39, 0.39, 0.39, 0.39, 0.39, 0.39, 0.39)	(1.99, 2.38, 6.76, 7.56, 1.99, 6.36, 2.78, 2.38, 3.18, 3.98)	(17.14, 21.01, 31.71, 35.58, 17.79, 32.06, 18.99, 17.6120.63, 23.66)
Scenario 2	20	(0.39, 0.39, 0.39, 0.39, 0.39, 0.39, 0.39, 0.39, 0.39, 0.39)	(1.99, 2.38, 6.76, 7.56, 1.99, 6.36, 2.78, 2.38, 3.18, 3.98)	(15.93, 19.79, 28.72, 31.86, 16.73, 29.57, 17.43, 16.35, 18.72, 21.80)
Scenario 3	30	(0.39, 0.39, 0.39, 0.39, 0.39, 0.39, 0.39, 0.39, 0.39, 0.39)	(1.99, 2.38, 6.76, 7.56, 1.99, 6.36, 2.78, 2.38, 3.18, 3.98)	(14.95, 18.75, 26.44, 29.10, 15.84, 27.58, 16.19, 15.32, 17.25, 20.31)
Scenario 4	40	(0.39, 0.39, 0.39, 0.39, 0.39, 0.39, 0.39, 0.39, 0.39, 0.39)	(1.99, 2.38, 6.76, 7.56, 1.99, 6.36, 2.78, 2.38, 3.18, 3.98)	(14.13, 17.87, 24.64, 26.96, 15.08, 25.96, 15.19, 14.47, 16.08, 19.09)
Scenario 5	50	(0.39, 0.39, 0.39, 0.39, 0.39, 0.39, 0.39, 0.39, 0.39, 0.39)	(1.99, 2.38, 6.76, 7.56, 1.99, 6.36, 2.78, 2.38, 3.18, 3.98)	(13.43, 17.10, 23.16, 25.23, 14.42, 24.58, 14.35, 13.74, 15.13, 18.08)
Scenario 6	60	(0.39, 0.39, 0.39, 0.39, 0.39, 0.39, 0.39, 0.39, 0.39, 0.39)	(1.99, 2.38, 6.76, 7.56, 1.99, 6.36, 2.78, 2.38, 3.18, 3.98)	(12.83, 16.42, 21.91, 23.79, 13.84, 23.40, 13.64, 13.12, 14.32, 17.20)

Note: Expressions in brackets correspond to the solution of retailers in sequence.

**Fig 4:**
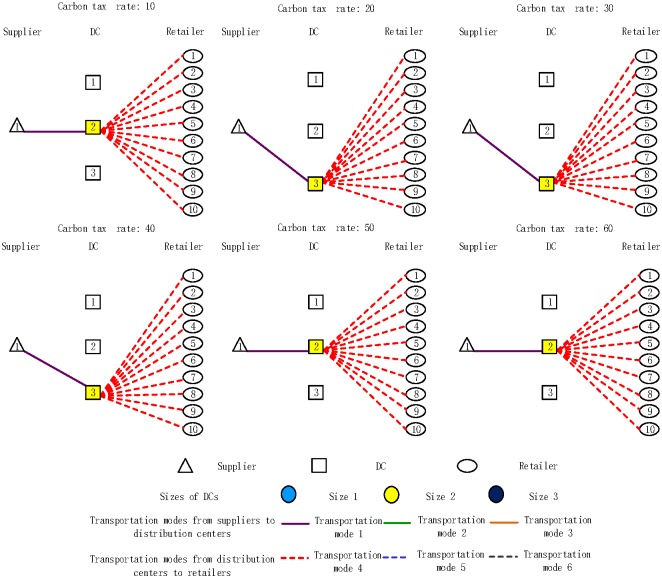
Distribution network design under different carbon emission tax rate scenarios.

The effect of various carbon emission rates on the distribution network design was also analyzed. The results presented in Fig 4. confirm that all opened DCs are consistently served by transport mode 1 and that all retailers are consistently served by transport mode 4. The number of opened DCs is equal to 1; it is stable, i.e., its size remains the same. However, the location of opened DCs varies with the increase in the carbon emission tax rate. For example, when the carbon emission tax rate is $10/ton, DC2 is selected to open, whereas for the carbon emission tax rate of $20/ton, the DC3 is selected.

### Effect of delay rate

The impacts of the delay rate parameter *λ* on the supply chain distribution network configuration and corresponding inventory control decisions are examined. Six different scenarios, i.e., *λ* = 0.04, 0.08, 0.12, 0.16, 0.20, and 0.24, are considered. The remaining parameters are set to be the same as those of the previous test.

**Fig 5:**
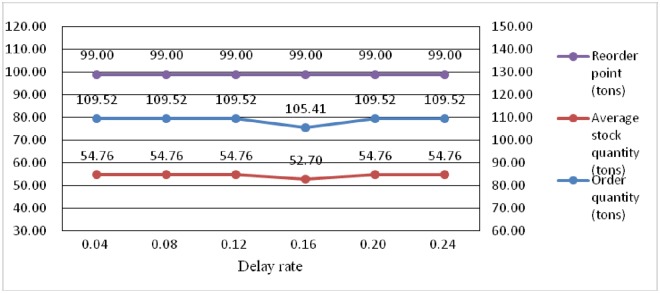
Comparative results of reorder point and order quantity and average shortage of opened DCs under different delay rates.

The analysis of the effects delay rate parameter on system performance of distribution networks is given in Table 5, clearly indicating that when the delay rate increases from 0.04 to 0.24, the replenishment cost of retailers gradually increases from $8,146.09 to $8,287.82. The inventory control parameters of the opened DCs and retailers corresponding to different delay rates are shown in Tables 6 and 7, respectively. According to Table 6 and Fig 5, the order quantity and reorder point of opened DCs remain the same during the increase of the delay rate. For example, the order quantity and reorder point at DC2 are 109.52 tons and 99.00 tons, respectively, when the delay rate is varied. Table 7 indicates that the replenishment lead time, reorder point, and order quantity of retailers continuously increase with the growth of delay rate. Based on Assumption A8, the delay rate of the replenishment lead time in retailers show significant impacts on inventory decisions at DCs and retailers. When the value of the delay rate parameter *λ* increases from 0.04 to 0.24, the replenishment lead time of retailers lengthens because of the shortage of DCs. This condition implies that the replenishment lead time increases with the increase of the delay rate. Consequently, the retailers must set a higher reorder point and larger order quantity to reduce the risk of shortage.

**Table 5:** Effects of delay rate parameter *λ* on performances of distribution network.

Delay rate	0.04	0.08	0.12	0.16	0.20	0.24
Fixed cost ($)	5,400.00	5,400.00	5,100.00	5,100.00	5,400.00	5,400.00
Carbon emission (ton)	134.09	134.35	138.15	138.40	134.42	134.68
Carbon emission cost ($)	4,022.70	4,030.50	4,144.50	4,152.0	4,032.60	4,040.40
Transport cost ($)	12,329.50	12,329.50	13,493.00	13,493.00	12,433.45	12,433.45
Replenishment cost at retailers ($)	8,146.09	8,174.80	8,203.27	8,231.58	8,259.76	8,287.82
Replenishment cost at DCs ($)	5,542.20	5,542.12	5,702.65	5,702.65	5,542.12	5,542.13
Total cost ($)	35,440.57	35,477.06	36,643.50	36,679.49	35,668.24	35,703.92

**Table 6:** Comparative results of DCs under different delay rates.

Delay rate	0.04	0.08	0.12	0.16	0.20	0.24
Reorder point	(0, 99.00, 0)	(0, 99.00, 0)	(0, 0, 99.00)	(0, 0, 99.97)	(0, 99.00, 0)	(0, 99.00, 0)
Order quantity	(0, 109.52, 0)	(0, 109.52, 0)	(0, 0, 105.40)	(0, 0, 105.40)	(0, 109.52, 0)	(0, 109.52, 0)
Average stock quantity	(0, 54.76, 0)	(0, 54.76, 0)	(0, 0, 54.76)	(0, 0, 54.76)	(0, 54.76, 0)	(0, 54.76, 0)

Note: Expressions in brackets correspond to the solution of DCs in sequence.

**Table 7:** Comparative results of retailers under different delay rates.

Scenarios	Delay rate	Optimal solutions		
		Replenishment lead time	Reorder point	Order quantity
Scenario 1	0.04	(0.13, 0.13, 0.13, 0.13, 0.13, 0.13, 0.13, 0.13, 0.13, 0.13)	(0.66, 0.79, 2.25, 2.51, 0.66, 2.12, 0.92, 0.79, 1.66, 1.32)	(14.90, 18.69, 26.17, 28.79, 15.80, 27.28, 16.11, 15.25, 17.17, 20.17)
Scenario 2	0.08	(0.26, 0.26, 0.26, 0.26, 0.26, 0.26, 0.26, 0.26, 0.26, 0.26)	(1.32, 1.59, 4.50, 5.03, 1.32, 4.24, 1.85, 1.59, 2.12, 2.65)	(14.93, 18.72, 26.31, 28.95, 15.82, 27.43, 16.15, 15.29, 17.21, 20.24)
Scenario 3	0.12	(0.40, 0.40, 0.40, 0.40, 0.40, 0.40, 0.40, 0.40, 0.40, 0.40)	(1.99, 2.38, 6.76, 7.56, 1.99, 6.36, 2.78, 2.38, 3.18, 3.98)	(14.95, 18.75, 26.44, 29.10, 15.84, 27.58, 16.19, 15.32, 17.25, 20.31)
Scenario 4	0.16	(0.53, 0.53, 0.53, 0.53, 0.53, 0.53, 0.53, 0.53, 0.53, 0.53)	(2.65, 3.18, 9.01, 10.07, 2.65, 8.48, 3.71, 3.18, 4.24, 5.30)	(14.98, 18.78, 26.58, 29.26, 15.86, 27.73, 16.23, 15.36, 17.29, 20.38)
Scenario 5	0.20	(0.67, 0.67, 0.67, 0.67, 0.67, 0.67, 0.67, 0.67, 0.67, 0.67)	(3.31, 3.97, 11.26, 12.59, 3.31, 10.60, 4.63, 3.97, 5.30, 6.62)	(15.00, 18.21, 26.72, 29.40, 15.87, 27.88, 16.27, 15.29, 17.33, 20.45)
Scenario 6	0.24	(0.80, 0.80, 0.80, 0.80, 0.80, 0.80, 0.80, 0.80, 0.80, 0.80)	(3.97, 4.77, 13.52, 15.11, 3.97, 12.72, 5.56, 4.77, 6.36, 7.95)	(15.02, 18.84, 26.86, 29.55, 15.89, 28.03, 16.31, 15.43, 17.38, 20.52)

Note: Expressions in brackets correspond to the solution of retailers in sequence.

**Fig 6:**
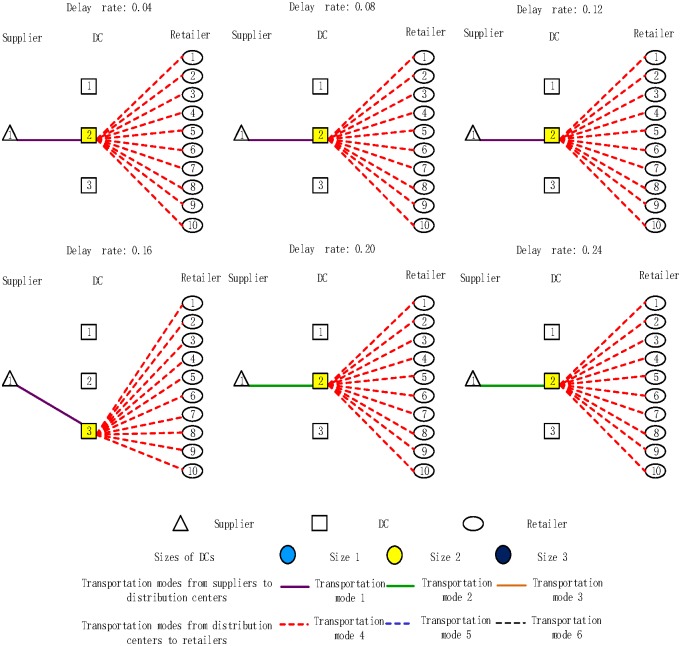
Distribution network design under six different delay rate scenarios.

In this study, six different delay rate scenarios were considered as shown in Fig 6 Obviously, the number of opened DCs is stable and equal to 1 when the delay rate increases from 0.04 to 0.24. Moreover, the sizes of opened DCs are always kept the same. The transport mode between the DCs and retailers remains the same, whereas the transport mode between the supplier and DCs changes when the delay rate increases from 0.09 to 0.24. The reason may be that the available discrete size series of DCs have different costs; the best choice of the size is a result of the balance between the transportation and inventory costs.

### Discussion and analysis

The aforementioned results indicate that an increase in the CO_2_ emission tax rate leads not only to a reduction in the total distribution network CO_2_ emissions but also to an increase in the total distribution network costs. The benefits gained from CO_2_ emission decrease by a charging policy that does not compensate for the extra costs of CO_2_ emission charges. Therefore, CO_2_ charging policy may contribute to a reduction of the CO_2_ emissions especially when CO_2_ is charged at a high rate. Moreover, the order quantities of opened DCs and retailers decrease with the increase of the CO_2_ emission tax rate. The reason is that CO_2_ emissions of inventory management are related to the average stock quantity of retailers and DCs. The preceding analysis implies that the policy of CO_2_ emission taxes will decrease the total CO_2_ emission of distribution network and induce supply chain managers to choose more green transport modes and corresponding inventory control policies.

The delay parameter *λ* at DCs shows a significant effect on the corresponding inventory control parameters of retailers such as replenishment lead time, reorder point, and order quantity of retailers. Specifically, the replenishment lead time, reorder point, and order quantity of retailers will increase with the increase of the delay rate at DCs, while the corresponding inventory control parameters of opened DCs are kept the same. An increase in the replenishment lead time of retailers will lead to higher-order quantity and reorder point.

Moreover, the location decision of DCs is insensitive to the changes of the delay rate. Specifically, the locations and sizes of opened DCs remain the same with the delay rate increase while the location of opened DCs varies with the increase of the CO_2_ emission tax rate.

## Conclusions

A new mathematical model to design a three-level distribution network design problem that considers the capacity constraints of DCs, transportation mode choices, and carbon emission tax policy was studied. Location, transportation, and inventory issues are included. The objective is to determine the distribution network decisions, such as the number of opened DCs, their locations, their sizes, and the assignments of each retailer and each opened DC, as well as the transportation mode choices. Furthermore, the inventory control decisions on the order quantity, reorder point, and service level at each retailer and opened DC are also determined. This study differs from previous related literature because the interaction effects of the two-echelon inventory control decisions have been analyzed. These effects include the shortage at DCs that possibly prolongs the replenishment lead time of retailers and the stock level of DCs being influenced by the demands of assigned retailers. In addition, the order quantity of products and reorder point for opened DCs and retailers were simultaneously determined.

An integrated inventory–location model was formulated as a mixed integer nonlinear programming problem with chance constraints corresponding to the capacity constraints of DCs and nonlinear objective function. LINGO software was used to solve the resulting MINLP problem. The effects of carbon emission tax rate and delay rate on the distribution network design, inventory control decisions, and total costs were discussed.

Future research directions are as follows: (1) investigating inventory sharing and lateral trans-shipments, which are becoming increasingly common in third-party logistics systems; (2) considering the extension of allowing the retailers to be sourced from multiple DCs; and (3) solving the presented model through heuristic or meta-heuristic algorithms.

## Supporting Information

S1 File Details of the derivation process of Eqs (5), (8) and (9). (DOCX)
