# Supplementary material for: Joint Optimization of Distribution Network Design and Two-Echelon Inventory Control with Stochastic Demand and CO2 Emission Tax Charges
Source: PLoS One. 2017 Jan 19;12(1):e0168526. doi: 10.1371/journal.pone.0168526 (PMC5245828; doi:10.1371/journal.pone.0168526)
Supplement: S1 File — (DOCX) [file pone.0168526.s001.docx]

# Appendixes

## Appendix 1

Constraint (5) is derived in detail, which represents the capacity restriction of DCs.
 $\Pr(R_{i}-D(LT_{i})+Q_{i}\leq u_{ia}C_{ia})\geq\delta\forall i=1,...,m$. (A-1)

The expression on the left side of Constraint (A-1) can be transformed into:

$$\begin{matrix} \Pr\left(R_{i}-D\left(LT_{i}\right)+Q_{i}\leq u_{ia}C_{ia}\right)=\Pr\left(D(LT_{i})\geq R_{i}+Q_{i}-u_{ia}C_{ia}\right) \\ \end{matrix}$$

$\begin{matrix} =\Pr\left(\frac{D(LT_{i})-\mu_{i}^{'}LT_{i}}{\sqrt{LT_{i}}\sigma_{i}^{'}}\geq\frac{R_{i}+Q_{i}-u_{ia}C_{ia}-\mu_{i}^{'}LT_{i}}{\sqrt{LT_{i}}\sigma_{i}^{'}}\right) \\ =\Pr\left(\frac{D(LT_{i})-\mu_{i}^{'}LT_{i}}{\sqrt{LT_{i}}\sigma_{i}^{'}}\geq\frac{LT_{i}u_{i}^{'}+Z_{\kappa_{i}}\sqrt{LT_{i}}\sigma_{i}^{'}+Q_{i}-u_{ia}C_{ia}-\mu_{i}^{'}LT_{i}}{\sqrt{LT_{i}}\sigma_{i}^{'}}\right) \\ =\Pr\left(\frac{D(LT_{i})-\mu_{i}^{'}LT_{i}}{\sqrt{LT_{i}}\sigma_{i}^{'}}\geq\frac{Z_{\kappa_{i}}\sqrt{LT_{i}}\sigma_{i}^{'}+Q_{i}-u_{ia}C_{ia}}{\sqrt{LT_{i}}\sigma_{i}^{'}}\right) \end{matrix}$ (A-2)

Hence, Constraint (A-1) can be re-arranged as follows:

$\Pr\left(\frac{D(LT_{i})-\mu_{i}^{'}LT_{i}}{\sqrt{LT_{i}}\sigma_{i}^{'}}\leq\frac{Z_{\kappa_{i}}\sqrt{LT_{i}}\sigma_{i}^{'}+Q_{i}-u_{ia}C_{ia}}{\sqrt{LT_{i}}\sigma_{i}^{'}}\right)\leq1-\delta$ (A-3)

This constraint can be reformulated as a deterministic nonlinear constraint (which assures that the probabilistic constraint is respected) as follows:

$\frac{Z_{\kappa_{i}}\sqrt{LT_{i}}\sigma_{i}^{'}+Q_{i}-u_{ia}C_{ia}}{\sqrt{LT_{i}}\sigma_{i}^{'}}\leq Z_{1-\delta}$ (A-4)

Therefore, Constraint ( A-4) can be simplified as follows:

$Q_{i}+Z_{\kappa_{i}}\sqrt{LT_{i}}\sigma_{i}^{'}+Z_{\delta}\sqrt{LT_{i}}\sigma_{i}^{'}\leq u_{ia}C_{ia}$ (A-5)

As mentioned above, Constraint (4) can be **equivalently transformed into** Constraint (5).

## Appendix 2

Expression (8) is derived, which represents the expected unfulfilled demand during order cycle.
 By definition, the expected unfulfilled demand of DC *i* can be obtained:

$E\left(LQ_{i}\right)=\int_{R_{i}}^{\infty} (x-R_{i})f_{i}\left(x\right)dx=\int_{R_{i}}^{\infty} xf_{i}\left(x\right)dx-\int_{R_{i}}^{\infty} R_{i}f_{i}\left(x\right)dx$, (A-6)

where$f_{i}\left(x\right)=\frac{1}{\sqrt{2\pi LT_{i}}\sigma_{i}^{'}}e^{-\frac{\left(x-LT_{i}u_{i}^{'}\right)^{2}}{2LT_{i}\left(\sigma_{i}^{'}\right)^{2}}}$ (A-7)

To compute this expression, a variable change as in Eq. (A-6) is first considered, logically satisfying Condition (A-8).

$y=\frac{\left(x-LT_{i}u_{i}^{'}\right)}{\sqrt{LT_{i}}\sigma_{i}^{'}}$ (A-8)

$x=y\sqrt{LT_{i}}\sigma_{i}^{'}+LT_{i}u_{i}^{'}$; $dx=\sqrt{LT_{i}}\sigma_{i}dy$ (A-9)

Replacing (A-8) with (A-6), the equation can be written as:

$$E\left(LQ_{i}\right)=\int_{R_{i}}^{+\infty} \left(x-R_{i}\right)f\left(x\right)dx$$

$$=\int_{\kappa_{i}}^{\infty} \frac{(y\sqrt{LT_{i}}\sigma_{i}^{'}+\mu_{i}LT_{i})}{\sqrt{2\pi LT_{i}}\sigma_{i}^{'}}e^{-\frac{y^{2}}{2}}\sqrt{LT_{i}}\sigma_{i}dy$$

$-\int_{\kappa_{i}}^{\infty} \frac{R_{i}}{\sqrt{2\pi LT_{i}}\sigma_{i}^{'}}e^{-\frac{y^{2}}{2}}\sqrt{LT_{i}}\sigma_{i}^{'}dy$ (A-10)

where$\kappa_{i}=\frac{\left(R_{i}-LT_{i}u_{i}^{'}\right)}{\sqrt{LT_{i}}\sigma_{i}^{'}}$ (A-11)

Rearranging Expression (A-10):

$\begin{matrix} E\left(LQ_{i}\right)=\int_{R_{i}}^{\infty} (x-R_{i})f\left(x\right)dx=\int_{\kappa_{i}}^{\infty} \frac{(y\sqrt{LT_{i}}\sigma_{i}^{'}+LT_{i}u_{i}^{'})}{\sqrt{2\pi}}e^{-\frac{y^{2}}{2}}dy-\int_{\kappa_{i}}^{\infty} \frac{R_{i}}{\sqrt{2\pi LT_{i}}\sigma_{i}^{'}}e^{-\frac{y^{2}}{2}}\sqrt{LT_{i}}\sigma_{i}^{'}dy \\ \end{matrix}$ (A-12)

where, . (A-13)

Finally, the mean of the unfulfilled demand can be computed as:

$$\begin{matrix} =\int_{\kappa_{i}}^{\infty} \frac{(y\sqrt{LT_{i}}\sigma_{i}^{'})}{\sqrt{2\pi}}e^{-\frac{y^{2}}{2}}dy+\int_{\kappa_{i}}^{\infty} \frac{LT_{i}u_{i}^{'}}{\sqrt{2\pi}}e^{-\frac{y^{2}}{2}}dy-\int_{\kappa_{i}}^{\infty} \frac{R_{i}}{\sqrt{2\pi}}e^{-\frac{y^{2}}{2}}dy \\ \end{matrix}$$

$$\begin{matrix} =\frac{-\sqrt{LT_{i}}\sigma_{i}^{'}}{\sqrt{2\pi}}\int_{\kappa_{i}}^{\infty} -ye^{-\frac{y^{2}}{2}}dy+\left(LT_{i}u_{i}^{'}-R_{i}\right)\int_{\kappa_{i}}^{\infty} \frac{1}{\sqrt{2\pi}}e^{-\frac{y^{2}}{2}}dy \\ \end{matrix}$$

$$=\frac{-\sqrt{LT_{i}}\sigma_{i}^{'}}{\sqrt{2\pi}}e^{-\frac{y^{2}}{2}}\left|_{\kappa_{i}}^{\infty}\right.+\left(LT_{i}u_{i}^{'}-R_{i}\right)\left(1-\int_{0}^{\kappa_{i}} \frac{1}{\sqrt{2\pi}}e^{-\frac{y^{2}}{2}}dy\right)$$

$$=\frac{-\sqrt{LT_{i}}\sigma_{i}^{'}}{\sqrt{2\pi}}\left(0-e^{-\frac{\kappa_{i}^{2}}{2}}\right)+\left(LT_{i}u_{i}^{'}-R_{i}\right)\left(1-\Phi\left(\kappa_{i}\right)\right)$$

$$=\frac{\sqrt{LT_{i}}\sigma_{i}^{'}}{\sqrt{2\pi}}e^{-\frac{\kappa_{i}^{2}}{2}}+\left(LT_{i}u_{i}^{'}-R_{i}\right)\left(1-\Phi\left(\kappa_{i}\right)\right)$$

(A-14)

## Appendix 3

Expression (9) is derived, which represents the variance of unfulfilled demand.
 By definition, the variance of unfulfilled demand at DC *i* can be written as follows:

$\text{Var}\left(LQ_{i}\right)=\int_{R_{i}}^{+\infty} \left[\left(x-R_{i}\right)-E\left(LQ_{i}\right)\right]^{2}f_{i}\left(x\right)dx$ $=\int_{R_{i}}^{+\infty} \left(x-R_{i}\right)^{2}f_{i}\left(x\right)dx-\left(\int_{R_{i}}^{+\infty} \left(x-R_{i}\right)f_{i}\left(x\right)dx\right)^{2}$ （A-15）

To compute this expression, a variable change as in Eq. (A-15) is considered first, thereby logically satisfying Condition (A-16).

$v=\frac{\left(x-\mu_{i}^{'}LT_{i}\right)}{\sqrt{LT_{i}}\sigma_{i}^{'}}$ (A-16)

$x=v\sqrt{LT_{i}}\sigma_{i}^{'}+\mu_{i}^{'}LT_{i}$; $dx=\sqrt{LT_{i}}\sigma_{i}^{'}dv$ (A-17)

Replacing (A-17) with (A-15), and considering Equation (A-11), the following can be obtained:

$$\text{Var}\left(LQ_{i}\right)=\int_{R_{i}}^{+\infty} \left(x-R_{i}\right)^{2}f_{i}\left(x\right)dx-\left(\int_{R_{i}}^{+\infty} \left(x-R_{i}\right)f_{i}\left(x\right)dx\right)^{2}$$

$$=\int_{\kappa_{i}}^{\infty} \frac{\left(v\sqrt{LT_{i}}\sigma_{i}^{'}-\kappa_{i}\sqrt{LT_{i}}\sigma_{i}^{'}\right)^{2}}{\sqrt{2\pi LT_{i}}\sigma_{i}^{'}}e^{-\frac{\left(v\sqrt{LT_{i}}\sigma_{i}^{'}\right)^{2}}{2LT_{i}\left(\sigma_{i}^{'}\right)^{2}}}\sqrt{LT_{i}}\sigma_{i}^{'}dv -\left(\int_{\kappa_{i}}^{+\infty} \frac{\left(v\sqrt{LT_{i}}\sigma_{i}^{'}-\kappa_{i}\sqrt{LT_{i}}\sigma_{i}^{'}\right)}{\sqrt{2\pi LT_{i}}\sigma_{i}^{'}}e^{^{-\frac{\left(v\sqrt{LT_{i}}\sigma_{i}^{'}\right)^{2}}{2LT_{i}\left(\sigma_{i}^{'}\right)^{2}}}}dv\right)^{2}$$

$$=LT_{i}\left(\sigma_{i}^{'}\right)^{2}\int_{\kappa_{i}}^{+\infty} \frac{\left(v-\kappa_{i}\right)^{2}}{\sqrt{2\pi}}e^{-\frac{v^{2}}{2}}dv$$

$$-LT_{i}\left(\sigma_{i}^{'}\right)^{2}\left(\int_{\kappa_{i}}^{+\infty} \frac{v}{\sqrt{2\pi LT_{i}}\sigma_{k}^{'}}e^{-\frac{v^{2}}{2}}dv-\int_{\kappa_{i}}^{+\infty} \frac{\kappa_{i}}{\sqrt{2\pi LT_{i}}\sigma_{i}^{'}}e^{-\frac{v^{2}}{2}}dv\right)^{2}$$

$$=LT_{i}\left(\sigma_{i}^{'}\right)^{2}\left[1-\Phi\left(\kappa_{i}\right)-\kappa_{i}\varphi\left(\kappa_{i}\right)+\left(\kappa_{i}\right)^{2}-\left(\kappa_{i}\right)^{2}\Phi\left(\kappa_{i}\right)\right]$$

$=LT_{i}\left(\sigma_{i}^{'}\right)^{2}\left[1+\kappa_{i}\varphi\left(\kappa_{i}\right)-\Phi\left(\kappa_{i}\right)+\left(\kappa_{i}\right)^{2}\Phi\left(\kappa_{i}\right)-\left(\kappa_{i}\right)^{2}\left(\Phi\left(\kappa_{i}\right)\right)^{2}-\left(\varphi\left(\kappa_{i}\right)\right)^{2}-2\left(\kappa_{i}\right)^{2}\varphi\left(\kappa_{i}\right)\Phi\left(\kappa_{i}\right)\right]$ (A-18)

Finally, the variance of unfulfilled demand of DC *i* can be simplified as follows:(A-19)
